# Inflammation and immune response in the development of periodontal disease: a narrative review

**DOI:** 10.3389/fcimb.2024.1493818

**Published:** 2024-11-29

**Authors:** Nansi López-Valverde, Norberto Quispe-López, José Antonio Blanco Rueda

**Affiliations:** Department of Surgery, University of Salamanca; Biomedical Research Institute of Salamanca (IBSAL), Salamanca, Spain

**Keywords:** inflammation, immune response, pyroptosis, inflammasome, periodontal disease

## Abstract

We present this critical review with the aim of highlighting the current status of periodontal diseases, focusing on the relevance of host modulating agents and immune pathways, in addition to new complementary therapeutic approaches for the treatment of these pathologies. Periodontal diseases are prevalent pathologies worldwide and the main cause of edentulism in the adult population. Their pathogenesis seems to be based on a dysbiosis of the oral microbiota that interacts with the host’s immune defenses and is responsible for the inflammatory/immune response, which would be modified by a number of conditions such as individual susceptibility, environmental and sociodemographic factors, certain systemic pathologies and the individual’s genetic condition, among others. Numerous studies have reported on the complex web of inflammatory mediators in periodontal disease and their role in tissue destruction as well as in homeostatic imbalance. Precisely, the role of epigenetics as a modifier of the host genetic condition has captured research attention in recent years. Therefore, this mini-review first discusses an updated etiological hypothesis of periodontal disease and the roles of certain cytokines in the immune response. In addition, the latest therapeutic trends with new developments and future perspectives are summarized.

## 1 Introduction

Periodontal diseases are considered a group of pathologies of inflammatory origin. Unlike periodontitis in which the lesions produced by the alteration of the dental supporting tissues are irreversible, gingivitis is reversible after resolutive treatment of gingival inflammation (Chapple et al., 2018).

Polymicrobial aggression, together with the host response and bacterial imbalance or dysbiosis, would be ultimately responsible for the establishment of the pathology (Hajishengallis et al., 2020 2000; Kinane et al., 2017); however, although more than 800 pathogens have been identified in different human biofilms (bacteria, archaea, protozoa, fungi and viruses) (Mosaddad et al., 2019; Antezack et al., 2023), it is still unknown which species cause the disease. This dysbiotic state, together with an exaggerated immune reaction, are major drivers of the inflammation and tissue damage detected in periodontal disease (Lamont and Hajishengallis, 2015). Additionally, individual susceptibility can be dictated by modifiers of the inflammatory response like environmental and sociodemographic factors, certain systemic pathologies, and the genetic condition of the host, among others, should be added (Scapoli et al., 2005; Shapira et al., 2005; Alawaji et al., 2022). This last situation is an interesting and intense focus of research trying to identify polymorphisms associated with different periodontal pathologies, and currently, it is considered that certain genes could be involved in periodontitis and that their genotypes could vary in different individuals or ethnic groups (Loos and Van Dyke, 2020 2000; Imamura et al., 2008). Another important aspect to consider would be epigenetic variants as modifiers of gene expression, acquired throughout life or inherited (Schulz et al., 2016; Jurdziński et al., 2020), although possible epigenetic implications in inflammatory pathologies have not been shown to be clinically relevant, and the role of epigenomic drugs is considered “potentially” novel in improving periodontal disease status (Barros et al., 2018 2000).

Bacterial dysbiosis induces exaggerated levels of inflammatory mediators, such as IL-1, prostaglandin E2 and tumor necrosis factor α (TNF-α) in subjects suffering from the disease, and initiates a cycle of exaggerated inflammatory response, aggravating tissue destruction (Gasmi Benahmed et al., 2022). Certain studies have shown that colonization by *Porphyromona gingivalis*, even at low levels, can alter the oral microbial homeostatic balance and trigger periodontal disease through inflammation and bone loss, produced by the dysbiotic state (Maekawa et al., 2014; Gasmi Benahmed et al., 2023). On the other hand, this homeostatic imbalance may undergo variations throughout the individual’s existence and be affected by conditions such as aging, epigenetic conditions and certain comorbidities that modify immune function (Abdulkareem et al., 2023).

The existence of dental biofilms (dental plaque) on the tooth surface is considered a natural phenomenon that helps to maintain the oral microbiota, preventing the invasion of exogenous species (Epsley et al., 2021), but dental plaque around the dental neck generates gingival inflammation and increased crevicular fluid, which is an excellent medium for the development of immunoglobulins, collagen degradation products, cytokines, serum proteins, etc. and above all of immune cells and desquamation of the internal epithelium of the periodontal pocket, together with the remains of gingival collagen degradation. In addition, the anoxic state of the environment, contribute to an increase in prevalence of anaerobic pathogens (Peng et al., 2024).

This mini-review aims to give a high-level overview on the current knowledge of the pathogenesis of periodontal disease, and the current evolution of its treatment, through new advances and emerging concepts, exposing different controversies and future perspectives. For more in-depth analyses of the literature, we direct readers to recent rigorous reviews (Marsh, 2010; Ray and Yung, 2018; Suárez et al., 2020).

## 2 Recently discovered inflammatory pathways in periodontitis

### 2.1 Resolvins

Granulocyte neutrophils or polymorphonuclear neutrophils (PMNs) are abundant in inflamed tissues and resolution of inflammation involves their elimination (Ariel et al., 2006); therefore, many therapeutic approaches are based on blocking the activation of inflammation, such as nonsteroidal anti-inflammatory drugs and tumor necrosis factor (TNF) inhibitors. Cyclooxygenase inhibitor drugs are a clear example of anti-inflammatory drugs that block prostaglandin synthesis (Kantarci et al., 2006 2000; Kirkwood et al., 2007 2000; Schonfeld, 2010). The lipoxins released by acetylsalicylic acid (ASA) and its synthetic derivatives, despite their toxic effect, favor the resolution of inflammation, especially by reducing the influx of neutrophils (Serhan, 2005). Precisely, a certain ω-3 fatty acid (eicosapentaenoic acid) is metabolized by cyclooxygenase-2 modified by ASA, giving rise to a small molecule (RvE1) capable of favoring the resolution of inflammation (Schwab and Serhan, 2006).

Resolvins are bioactive products of ω-3 fatty acids that counteract proinflammatory signals by retaining leukocyte recruitment. Hasturk et al. in an *in vivo* study suggested that RvE1, in topical application in rabbits with periodontitis, protected against inflammation-induced bone and soft tissue loss (Hasturk et al., 2006). Similarly, Lee et al (Lee et al., 2016). demonstrated in a rat model with ligation periodontitis that topical treatment with RvE1 prevented bone loss by reducing osteoclast density and inflammation-related gene expression, along with modifications in subgingival microbiota and bacterial growth conditions. This is despite the important role of inflammation in this regard. Hasturk et al. reported in an *in vivo* study, regeneration of hard and soft tissues, destroyed by inflammatory diseases by monotherapy of activation of inflammation resolution pathways with RvE1, obtained from ω-3, demonstrating the role of local inflammation in tissue destruction (Hasturk et al., 2007).

### 2.2 Inflammasomes, pyroptosis and their role in periodontal pathology

Inflammasomes are protein complexes located in the cell cytoplasm that act as sensors and mediate the development of inflammation (Paerewijck and Lamkanfi, 2022). More and more studies are investigating biomarkers of periodontitis (Isaza-Guzmán et al., 2017; Isola et al., 2022) and there is increasing evidence that inflammasomes are involved in the periodontal immune response, controlling invading microorganisms (Li et al., 2021b; Sordi et al., 2021). It is known that excessive activation of inflammasomes leads to inflammatory dyscontrol (by the release of proinflammatory cytokines IL-1β, IL-18), cytokine storm and tissue damage, and that patients with periodontal pathologies present elevated levels of certain inflammasomes in saliva, proportional to the severity of the disease. On the other hand, the oral microbiota deregulates the tissue expression of the NLRP3 inflammasome, which aggravates periodontal inflammation (Zhao et al., 2022; Didilescu et al., 2024). Polymerase chain reaction (PCR), used for the detection of periodontal pathogens in aggressive periodontitis, showed an increase of IL-1β, produced by macrophages and monocytes, in early stages of inflammation, which would signify the important role of local inflammation on systemic inflammation (Noack et al., 2001; Ebersole et al., 2002; Salzberg et al., 2006; Lopez-Castejon and Brough, 2011).

Periodontal pathogens, such as *P. gingivalis* and *Fusobacterium nucleatum*, are known to activate the canonical NLRP3 inflammasome (Aral et al., 2020). Activated canonical NLRP3 directly stimulates caspase-1 (a protein mediating the processes of programmed cell death, or apoptosis), leading to maturation and secretion of proinflammatory cytokines (Man et al., 2017).

The cytokine IL-1β is instrumental in the development of periodontal pathology and it is well known that NLRP3 is involved in its maturation (Chen Y. et al., 2021; Li et al., 2021b). Thus, the abnormal activation or overexpression of NLRP3 in osteoclasts, osteoblasts, fibroblasts, and immune cells is considered to play a critical role in the pathogenesis of periodontal disease (Zhao et al., 2022).

**Figure 1:**
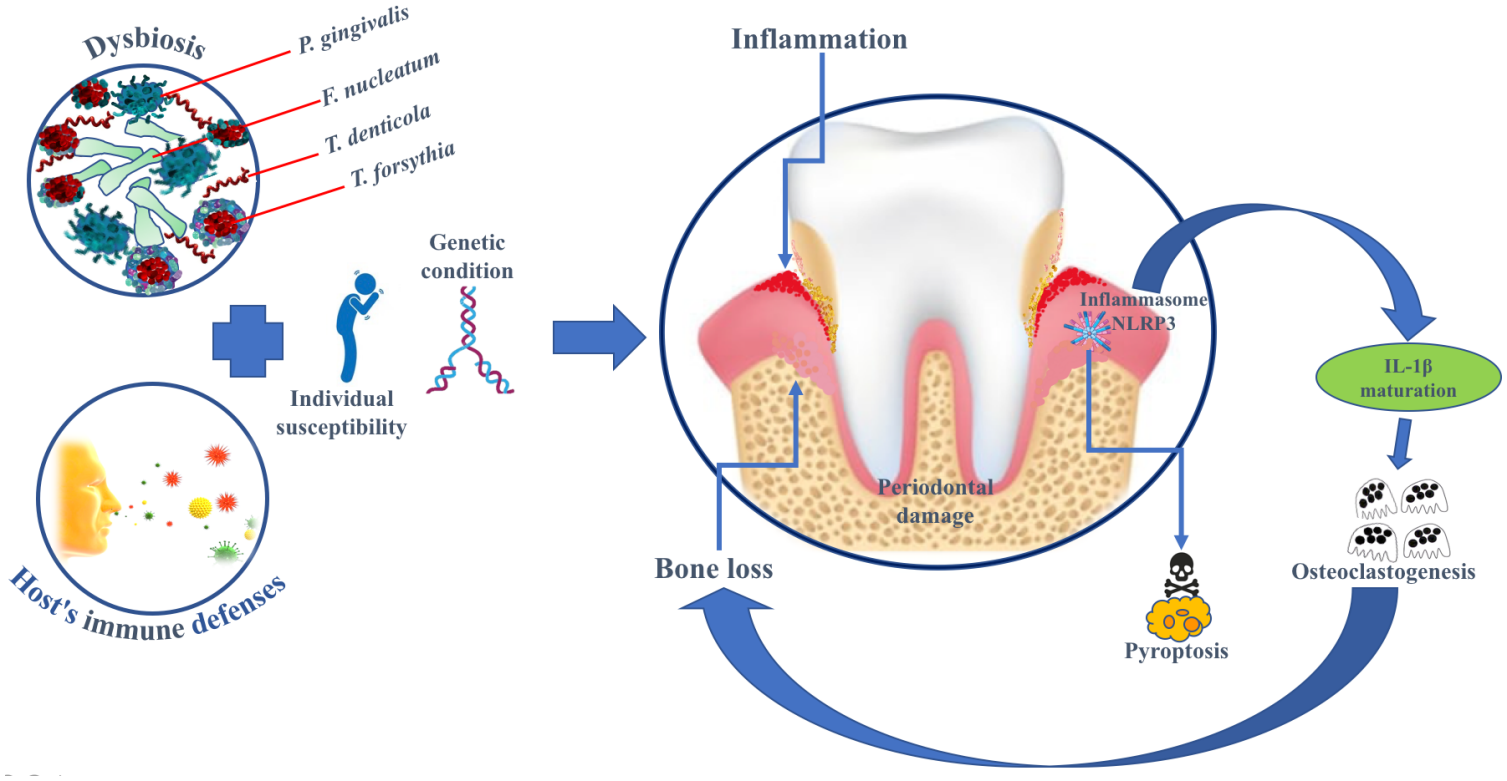
Diagram of causative-predisposing agents and the role of inflammasomes in pyroptosis, osteoclastogenesis and bone loss in periodontal disease.

Osteoclasts play an important role in bone loss processes and excessive osteoclastic activity leads to bone destructive pathologies. IL-1β has been observed to potentiate osteoclastogenesis through extracellular matrix degradation (Zhou et al., 2022; Otsuka et al., 2023) (**Figure 1**).

Using a mouse model of induced periodontitis, Chen et al (Chen Y. et al., 2021), were able to suppress alveolar bone loss using a specific NLRP3 inhibitor, which also reduced IL-1β activation and osteoclast differentiation. The NLRP3 inflammasome has also been observed to play an important role in osteoclastogenesis during aging (Zang et al., 2020). A recent review conducted by Bakhshi et al (Bakhshi and Shamsi, 2022). reported that the NLRP3 inflammasome may be involved in the pathogenesis of several inflammatory and autoimmune diseases such as type 2 diabetes mellitus, and that inhibition of this inflammasome could be a useful treatment option for inflammatory diseases, by reducing the production of the cytokine IL-1β. Together, this supports targeting the inflammasome to treat multiple inflammatory diseases.

Osteoblasts participate in bone mineralization (Zhao et al., 2014) but, when infected by certain gram-negative pathogens, such as *Aggregatibacter actinomycetemcomitans*, they generate IL-1β and programmed cell death, mediated by NLRP3 activation (Herbert et al., 2016; Wang et al., 2017). Reactive oxygen species (ROS) are a determining factor in NLRP3 activation and certain research has demonstrated the role of oxidative stress (OS) in the pathogenesis of periodontal disease and periodontal tissue damage, as well as the beneficial role of antioxidant therapies (Sczepanik et al., 2020 2000; Liu et al., 2020; López-Valverde et al., 2024). Bone regeneration is a complex process in which, in addition to metabolism, differentiation and cell migration, the immune system is involved (Ye et al., 2021). This system is associated with bone loss and it has been shown that inflammatory stimuli, due to an immune imbalance, can cause an alteration of bone turnover through osteoclastic differentiation, together with a slowing of osteoblastic differentiation, which could generate different bone metabolic pathologies (Xiong et al., 2022). The bone formation/resorption balance goes through the regulation of pyroptosis and it is known that NLRP3 is able to produce IL-1β and trigger pyroptosis in response to molecular patterns associated with periodontal pathogens; however, an inappropriate activation of the inflammasome, may generate an environment prone to inflammation and massive cell destruction, as occurs in the bone destruction characteristic of periodontitis (Chen Q. et al., 2021). Thus, pyroptosis and certain cytokines, such as IL-1β, are maintainers of homeostasis and drivers of the individual’s innate immune response, shaping his or her adaptive immunity (Li and Jiang, 2023).

On the other hand, the important role of periodontal ligament fibroblasts in the regenerative functions of alveolar bone and in inflammation through the production of proinflammatory cytokines that damage the periodontal ligament is noteworthy (Isaka et al., 2001). Gram-negative bacterial lipopolysaccharide (LPS) is a major component of the outer membrane and plays a key role in host-pathogen interactions with the innate immune system. *In vitro* studies in human periodontal ligament cells have shown that LPS would be able to trigger pyroptotic cell death in periodontal ligament cells and promote the generation and secretion of proinflammatory cytokines (Zhang et al., 2021). Zhang et al. in a recent study in a rat model reported a potent virulence factor secreted by certain periodontal pathogens, such as *F. nucleatum* and *P. gingivalis*, that would be involved in the damage of gingival epithelium, periodontal ligament, alveolar bone and other peripheral tissues and would be able to trigger the activation of NLRP3, the neutralization of which would be instrumental in the treatment of periodontitis (Zhang et al., 2024).

However, there are few studies linking NLRP3 to periodontal ligament fibroblasts, which would warrant further study. PMNs are the most abundant leukocyte species in inflamed periodontal tissues (Ginesin et al., 2023). Dysfunction of PMNs is determinant in periodontitis and related comorbidities. Inflammatory persistence of periodontitis can lead to aberrant neutrophil activation and sustained release of proinflammatory mediators, resulting in tissue damage, bone resorption, and progression of periodontal disease (Bassani et al., 2023). The infiltration of PMNs at the periodontal lesion site is dependent on the expression of NLRP3 (Han et al., 2022). Surlin et al. in a study of 62 participants to evaluate the impact that periodontal disease and chronic hepatitis C might have on NLRP3 levels, along with increased local inflammatory reaction with periodontal clinical consequences, found significantly elevated levels for NLRP3 in the hepatitis C and periodontitis group compared to the non-periodontitis groups. Furthermore, they found a positive correlation of NLRP3 levels, together with certain metabolic parameters, including glucose, aspartatetransferase and alaninetransferase levels, demonstrating that chronic hepatitis C and periodontal disease could significantly influence the up-regulation of NLRP3 and its components, possibly contributing to an increased local inflammatory reaction (Surlin et al., 2021).

Cellular self-destruction contributes to homeostasis and to the defense of the human organism against pathogen aggression. One of the mechanisms of self-destruction, apoptosis, is described as a programmed and active process that prevents inflammation, unlike necrosis, and is characterized as a passive and accidental cell death (Fink and Cookson, 2005). Another mechanism of programmed cell death, pyroptosis, is characterized by cell swelling and subsequent rupture of the cytoplasmic membrane, releasing a large number of molecules that trigger a strong inflammatory response, with subsequent recruitment of immune system cells (Yu et al., 2021). In inflammatory pathologies, such as periodontal diseases, the responsible pathogens can trigger pyroptosis of host cells through NLRP3 activation. This would result in the subsequent release of proinflammatory cytokines, increasing the host immune response and thus tissue destruction (Chen et al., 2024).

Several studies have highlighted the role of pyroptosis in periodontal disease, demonstrating that LPS from *P. gingivalis* would be able to induce gingivitis, destroy the epithelial connection and increase the expression of pyroptosis-associated proteins (Li et al., 2021a; Li YY. et al., 2021; Lv et al., 2021; Yang et al., 2021). Pan et al. found bidirectionality in the up-regulation of IL-1β in PMNs as a mechanism of cell death in periodontitis, underlining the importance of this finding in the pathogenesis of the disease (Pan et al., 2023).

## 3 Periodontal therapeutics

Although periodontal diseases are highly prevalent and functionally and esthetically disabling, there is a lack of unified criteria for their diagnosis. Understanding the pathognomonic mechanisms that cause them is key to developing preventive measures and effective treatments, as traditional surgical treatments are often ineffective, especially in patients with exaggerated immune responses.

### 3.1 Inflammasome inhibitor drugs

NLRP3 is known to be involved in a wide variety of infections directly related to inflammatory and degenerative pathologies (Stout-Delgado et al., 2016; Shen et al., 2020; Liu et al., 2022; Ye et al., 2023), but drugs directed against NLRP3 are scarce. Recently, Ye et al (Ye et al., 2023). have proposed peptides as more suitable remedies against small molecules, with the advantage of being more potent, less toxic and having fewer unwanted effects. Coll et al (Coll et al., 2015). proposed a specific small molecule inhibitor of NLRP3, at reduced doses, which has a potent action in numerous pathologies of inflammatory origin. Subsequently, other studies have shown that MCC950 would enhance neuroinflammation-related neurogenesis by disrupting NLRP3 activation (Gordon et al., 2018). All this would lead one to believe that MCC950 could suppress NLRP3 (Tang et al., 2017); however, despite undergoing phase II clinical trials, the research was abandoned due to liver damage in some cases (Mangan et al., 2018). Although not evaluated in periodontal disease models, Jiang et al (Jiang et al., 2017), in autoinflammatory syndrome mouse models, identified a small molecule, CY-09, capable of specifically inhibiting NLRP3 and suppressing IL-1β production.

### 3.2 Systemic antibiotics

Systemic antibiotics such as metronidazole, amoxicillin or ciprofloxacin are still used in the treatment of periodontitis and are capable of eliminating or greatly reducing the main periodontal pathogens (Slots, 2020 2000), but the adverse reactions and side effects of this type of treatment make it necessary to develop new therapies that do not have these drawbacks.

### 3.3 Semi-synthetic derivatives of Artemisinin

Artesunate, a natural peroxide, derived from the herb Artemisia Annua, has been proposed for its demonstrated anti-inflammatory and immunomodulatory effects in different bone pathologies (Zhang, 2020). Huang et al (Huang et al., 2022). demonstrated its efficacy in an osteoporotic murine model, in which it produced a marked increase in alkaline phosphatase activity and osteogenesis-related molecules. Recently, a study by Wang et al (Wang et al., 2023). in rats with ligation-induced periodontitis, showed that artesunate was able to reduce alveolar bone loss generated by periodontitis and suppress osteoclastogenesis, as well as stimulate osteogenic potential and reduce cytokine expression under inflammatory conditions. Other investigations have also highlighted the therapeutic potential of artesunate on inflammatory destruction, due to its endoperoxide group (Su et al., 2021; Wang et al., 2022).

### 3.4 Sulfonylureas

Glyburide is a hypoglycemic sulfonylurea used for the treatment of type 2 diabetes mellitus that has also demonstrated anti-inflammatory capacity, both in humans and in animal models. While its ability to suppress IL-1β activation and release is unclear, it seems likely that this ability would only be present in the absence of pyroptosis (Roglic and Norris, 2018; Zahid et al., 2019). In addition, its ability to inhibit inflammatory cell infiltration, osteoclast formation and bone resorption has been demonstrated in rats with experimental periodontitis, suggesting that glyburide may be therapeutically useful as a treatment for periodontal diseases (Kawahara et al., 2020).

These therapeutic targets against inflammasomes are promising candidates, but further research must be conducted to ensure efficacy against periodontal disease and safety in humans.

## 4 Control of immune response; new therapies

The role of cell destruction and cell death in periodontitis remains largely unknown and therefore, necessary to further explore this aspect to clarify its implications.

It has been reported that both periodontitis and rheumatoid arthritis may share the presence of periodontal pathogens that promote protein shedding, resulting in anti-citrullinated protein antibodies, a typical trigger for autoimmune pathologies (Corrêa et al., 2019). A meta-analysis by Zamri and de Vries (2020) investigated the duration of anti-TNF-α treatment on periodontal clinical parameters, finding that treatments of less than 6 months were beneficial, whereas those of more than 6 months were associated with higher gingival indices and bleeding on probing, possibly due to the development of anti-drug antibodies. Similarly, another recent review (Inchingolo et al., 2023) that investigated the effect of antirheumatic drugs on periodontal indices and cytokine levels in periodontitis demonstrated beneficial effects of these drugs on clinical and immunological parameters of the periodontium. It is known that B lymphocytes are present in areas of chronic periodontal inflammation, stimulating osteoclasts through the genesis of IL-6, IL-17 and Receptor Activating Nuclear Factor κ B Ligand (RANKL) and that patients treated with B-lymphocyte blocking drugs presented less aggressive forms of periodontitis (Coat et al., 2015); however, the use of this type of drugs is associated with a number of side effects, such as skin reactions, cardiac failure and hepatotoxicity, which limits their usefulness in the treatment of periodontal pathologies (Zamri and de Vries, 2020).

Probiotics and ω-3 fatty acid supplements are being promoted as potential therapeutic candidates in the treatment of inflammatory pathologies. An extensive review by Homayouni et al (Homayouni Rad et al., 2023). highlighted the important role of probiotics as antioxidant producers and plaque formation preventive agents. Allaker and Stephen (2017) advocated them as modulators of host dysbiosis and immune-inflammatory pathways, thus reducing the destructive capacity of periodontal disease. However, attention has been drawn to the potential risks of prolonged consumption in subjects with a weakened immune system (Tegegne and Kebede, 2022).

The reduction of periodontal inflammation with ω-3 fatty acids, as a modulatory therapy and observing its effects on pocket probing depth and clinical attachment level, has been carried out in different studies as an adjunct to surgical therapy, demonstrating its efficacy, despite scarce clinical evidence (Van Ravensteijn et al., 2022).

Statins are cholesterol-lowering drugs with anti-inflammatory, anticoagulant and antioxidant effects (Biasucci et al., 2010), in addition to other benefits on endothelial cell function and modulation of the inflammatory response. In the treatment of periodontitis, they have been used in preclinical studies, locally in periodontal pockets, observing an increase in antioxidants, together with an increase in anti-inflammatory mediators and a reduction in bone resorption (Sousa et al., 2016).

**Figure 2:**
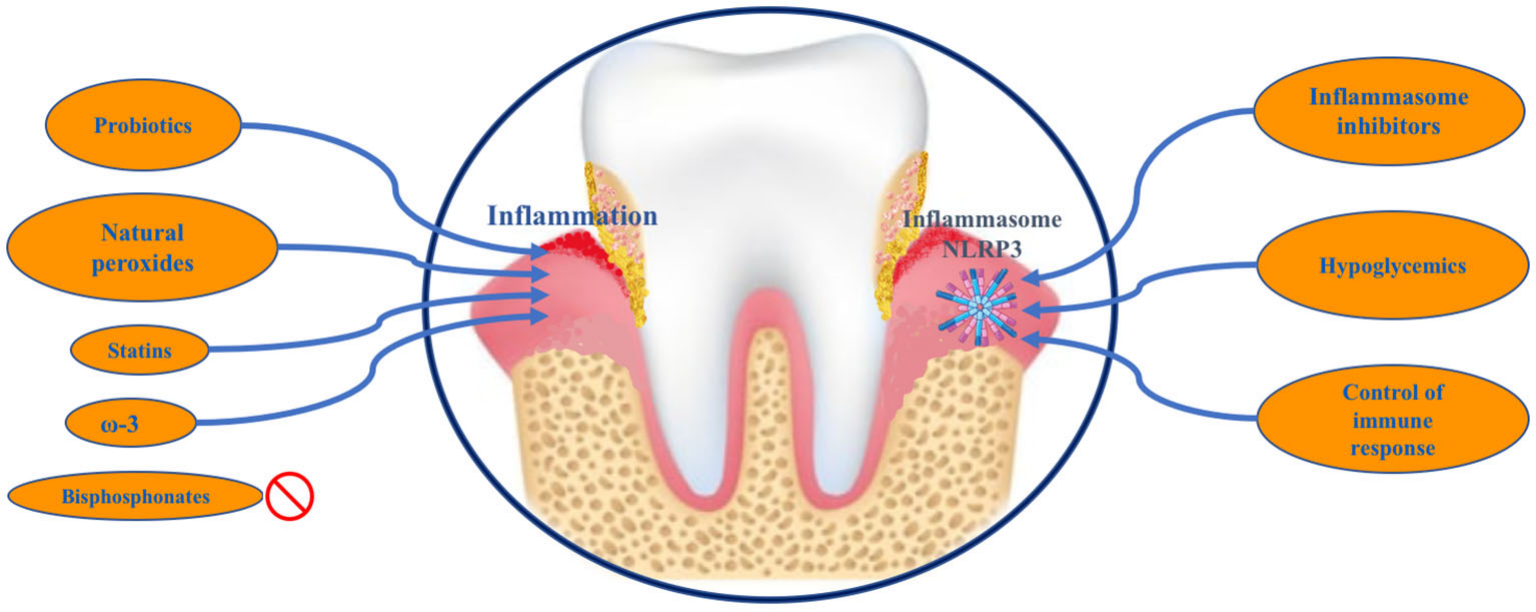
Representative diagram of future therapeutic targets in periodontal disease.

The use of bisphosphonates in the treatment of periodontitis, despite their inhibitory effect on bone destruction, is controversial, mainly due to the side effect of mandibular osteonecrosis (Aguirre et al., 2021) (**Figure 2**).

## 5 Conclusions and perspectives

Although dysbiosis is the primary driver of periodontal disease, the NLRP3 inflammasome, which mediates the maturation of the cytokine IL-1β, plays a crucial role in its pathogenesis. The limited efficacy of traditional surgical treatments in some cases highlights the potential of alternative therapies, including systemic antibiotics, NLRP3 inhibitors and anti-inflammatory drugs. However, their use is often hampered by adverse reactions and other drawbacks. Although treatments such as ω-3 fatty acid supplements, probiotics, statins and bisphosphonates offer some immune modulation, their long-term use carries potential risks. These challenges underscore the need for further research and development of new therapeutic options.
